# Impact of COVID-19 on the mental health of older adults, people with dementia, and carers in lower- and middle-income countries: An international qualitative study

**DOI:** 10.1192/j.eurpsy.2023.876

**Published:** 2023-07-19

**Authors:** L. Ssenyonjo, I. Ddumba

**Affiliations:** 1 Research & Innovation, African Research center 4 Ageing & dementia; 2 Nursing, Victoria University, Kampala, Uganda

## Abstract

**Introduction:**

The Covid-19 pandemic has exacerbated mental health problems in many countries, yet little evidence has focused on older adults.

**Objectives:**

The aim of this study was to qualitatively explore the impact of the pandemic on the mental health and well-being of older adults living in Uganda

**Methods:**

Semi-structured interviews with older adults, family carers, and people living with dementia, plus focus groups with care professionals were conducted remotely via the telephone. Data were collected at two time points between March and July 2021. Non-professionals were asked about their experiences of the pandemic and their mental well-being. Data were analysed using thematic analysis. All transcripts were also translated into English and a selection were second-coded by the another team.

**Results:**

A total of 30 interviews were conducted with older adults, people with dementia, and unpaid carers participating at baseline (n=30). Using inductive thematic analysis, we generated three overarching themes: Mental health needs overridden by need for basic necessities; Social isolation; Increased worry about restrictions and pandemic in dementia. For most people, limited access to basic necessities, including food, featured more prominently in responses than any direct acknowledgement of how the pandemic has affected their mental well-being. Participants were upset and worried about being socially isolated, with carers concerned about the welfare of many people with dementia and often feeling emotionally exhausted.

**Conclusions:**

Older adults, carers, and people living with dementia in Uganda not only require support to cope with the mental health impact of the pandemic, but most importantly require improved financial governmental support to be able to access sufficient food and other basic necessities, as a group their health is poor and associated risk of deterioration high

**Disclosure of Interest:**

None Declared

